# Vegetative growth drives the negative effects of an invasive species on resident community diversity and is not limited by plant–soil feedbacks: A temporal assessment

**DOI:** 10.1002/ece3.70070

**Published:** 2024-07-22

**Authors:** Emily M. Holden, Karina Salimbayeva, Charlotte Brown, Gisela C. Stotz, James F. Cahill

**Affiliations:** ^1^ Department of Biological Sciences University of Alberta Edmonton Alberta Canada; ^2^ Départment de Biologie Université de Sherbrooke Sherbrooke Quebec Canada; ^3^ Centro de Investigación Para la Sustentabilidad, Facultad de Ciencias de la Vida Universidad Andrés Bello Santiago Chile

**Keywords:** biodiversity, community assembly, conservation, invasion, smooth brome, soil biota

## Abstract

Many pathways of invasion have been posited, but ecologists lack an experimental framework to identify which mechanisms are dominant in a given invasion scenario. Plant–soil feedbacks (PSFs) are one such mechanism that tend to initially facilitate, but over time attenuate, invasive species' impacts on plant diversity and ecosystem function. PSFs are typically measured under greenhouse conditions and are often assumed to have significant effects under field conditions that change over time. However, direct tests of PSFs effects in natural settings and their change over time are rare. Here we compare the role of PSFs with the effects of biomass in limiting the dominance of an invasive species and impacts on resident species diversity. We characterized the effects of the invader *Bromus inermis* (Leyss.) on native plant communities over time and measured changes in its conspecific PSFs and vegetative growth to understand their integrated effects on community diversity. To do so, we combined data from a 6‐year field study documenting the rate and impacts of invasion with a short‐term greenhouse experiment quantifying PSF as a function of time since invasion in the field. We found that the nature and strength of *B. inermis* PSFs did not change over time and were not mediated by soil microbial communities. Though PSFs impacted *B. inermis* reproduction, they did not sufficiently limit vegetative growth to diminish the negative impacts of *B. inermis* biomass on native species. *B. inermis* experienced the full strength of its negative PSFs immediately upon invasion, but they were ineffective at reducing *B. inermis* vigor to facilitate the recovery of the native plant community. We recommend that conservation efforts focus on limiting *B. inermis* vegetative growth to facilitate community recovery.

## Graphical abstract

We investigated the role of plant–soil feedbacks (PSFs) and biomass in limiting the dominance of the invasive species *Bromus inermis*. We found that though PSFs affected *B. inermis* reproduction, they did not reduce its vegetative growth or its negative effects on native species diversity over time. We suggest conservation efforts should prioritize controlling *B. inermis* vegetative growth to aid native community recovery.

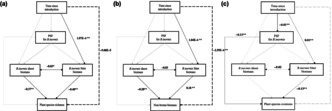

## 1 INTRODUCTION

Invasive plant species are widely recognized as a major threat to native biodiversity and ecosystem stability (IPBES, 2023). They can become dominant in communities and displace resident species through multiple mechanisms, including increased competition via enhanced vegetative growth. The dominance of invasive species in a new range can result from the alteration of the soil environment which may also cause changes in the abundance and identity of soil biota (Callaway et al., 2008; Ehrenfeld, 2003; Lai et al., 2018; MacDougall & Turkington, 2005). In the early stages of the invasion process, many invasive plant species modify soil properties to their advantage, creating positive conspecific plant–soil feedbacks (PSFs) that destabilize coexistence and may lead to diversity loss (Klironomos, 2002; but see Stotz et al., 2018). However, any such advantage exerted by invasive species in the new range may attenuate over time as it accumulates pathogens or detrimental allelochemicals (da Silva et al., 2017; Smith‐Ramesh & Reynolds, 2017). This would shift PSFs to become negative and suppress the growth of invasive plant species when growing in their own soil (Diez et al., 2010; Dostál et al., 2013; Flory & Clay, 2013; Hawkes et al., 2007; but see Day et al., 2015). Though these changes in the strength and direction of PSFs over time are presumed to be important, they are rarely documented, and their consequences are rarely assessed under natural field conditions.

There is a large focus on the role of soil microbes in PSF (e.g., Bever, 1994; van der Putten et al., 2013), but plants can also alter the abiotic properties of the soil through cascading effects (e.g., Goldberg, 1990; Tilman, 1982, 1988). For example, plants can modify soil nutrient availability and induce PSFs through local nutrient depletion or alter nutrient cycling via litter deposition (Fujii et al., 2018; Hobbie, 2015; Kulmatiski et al., 2017). Further, soil biota may interact with available nutrients (see Revillini et al., 2016; Thrall et al., 2007; also Chagnon et al., 2018) or soil characteristics (e.g., soil texture, temperature, etc.; see Bezemer et al., 2006; Heinze et al., 2017) to induce population responses unique to a given locale (Smith‐Ramesh & Reynolds, 2017). PSF context‐dependency is also influenced by legacies of previously different abiotic conditions (Crawford & Hawkes, 2020; de Vries et al., 2023). Thus, to better understand the relative effect of soil microbes under natural field conditions, it is critical to include the measures of the abiotic PSFs when exploring PSF effects. This is especially important when considering local management actions, as the context‐dependency of PSFs may change the outcome away from the management target.

PSFs influence plant growth in greenhouse settings but may be unimportant for determining population dynamics in the field. When grown in a community context, the effects of negative conspecific PSFs may not be strong enough to slow the invader's population growth and facilitate the recovery of the resident community (see Stotz et al., 2019). Thus, in addition to determining whether conspecific PSFs have *significant* effects on invasion, we must also determine whether their effects are *important* relative to other simultaneously operating processes to fully understand PSFs' potential to mitigate the detrimental effects of invasive species (see Karst et al., 2023). A second limitation may stem from the fact that most studies compare processes in invaded and uninvaded areas rather than focusing on invasion temporal dynamics, even when the mechanisms facilitating invasive species' initial dominance may differ from those supporting long‐term dominance. Negative PSFs experienced by the invading species could become a stabilizing mechanism that reduces invasive species dominance and facilitates resident species recovery over time, which is of great conservation interest (Bever, 1994, 2003; Dostál et al., 2013; Hawkes et al., 2007). But, despite its significant management implications, the role of PSFs in nature is rarely tested (though see Beckman et al., 2023 and Forero et al., 2019).

*Bromus inermis* Leyss. is a perennial grass native to central Eurasia and a prolific invader of North American grasslands, rapidly spreading and resulting in declines in native species diversity (Bennett et al., 2014; Mamet et al., 2017), which makes it of conservation concern. *B. inermis* reproduces both sexually through seeds and vegetatively via rhizomes, which facilitates its rapid spread (Otfinowski et al., 2007). It is known that *B. inermis* experiences negative PSFs when grown in its own soil (Stotz et al., 2018) and that it modifies soil microbial communities via its litter accumulation (Piper, Lamb, & Siciliano, 2015; Piper, Siciliano, Winsley, & Lamb, 2015; Stotz et al., 2019). Litter accumulation is speculated to increase *B. inermis* invasive potential (Vinton & Goergen, 2006; but see Carrigy et al., 2016). Yet, it is unknown whether conspecific PSFs can outweigh other potential negative effects of *B. inermis*, such as its high stand density and litter accumulation (Fink & Wilson, 2011; Stotz et al., 2016), to allow for the recovery of the resident community, motivating the current study. Synthetic modeling techniques such as structural equation models (SEM; Grace, 2006) are very well suited to identify and isolate the effect of different factors, and quantify their relative contributions To this end, we use an experimental framework to discern the contributions of two potential pathways (vegetative biomass vs. PSFs) to invasion dynamics in a grassland system.

In this study, we assess whether conspecific PSFs are important in field settings relative to other factors in mitigating *B. inermis*' impacts on resident communities under natural field conditions. To do so, we used data from a long‐term study tracking *B. inermis* invasion that captured changes in community characteristics in areas with different *B. inermis* residence time. We first estimated the time since invasion along the encroachment front to capture the temporal dynamics of *B. inermis* invasion. Then, using field‐collected soils, we performed a paired greenhouse experiment and field sampling to answer the following questions:

1. To what degree do conspecific *B. inermis* PSFs have a greater negative impact on its growth and reproduction over time?
2. Does the impact of *B. inermis* invasion on native plant communities change over time?
3. How does the relative contribution of PSFs compare to other factors to potentially limit the impact of *B. inermis* on native plant diversity?

## 2 MATERIALS AND METHODS

### 2.1 Study species

*Bromus inermis* Leyss. (smooth brome) is a perennial cool‐season grass native to central Eurasia that was introduced to Canada as a forage crop in the late 1800s (Otfinowski et al., 2007). Following its introduction, *B. inermis* escaped from cultivation and has since been ranked as one of the most harmful invasive species in Canada (Catling & Mitrow, 2005). It establishes within varied habitats but is most detrimental to the diversity of native grasslands (Carrigy et al., 2016; Otfinowski et al., 2007; Stacy et al., 2005; Stotz et al., 2019). Where it invades, *B. inermis* often forms dense, large patches (~60% cover), decreasing light availability and soil moisture (Bennett et al., 2014; Fink & Wilson, 2011). *B. inermis* also increases soil nutrient availability through litter accumulation, which alters microbial communities (Piper, Lamb, & Siciliano, 2015; Piper, Siciliano, Winsley, & Lamb, 2015).

### 2.2 Estimating time since invasion

To examine the temporal dynamics of PSFs, we focused on *B. inermis* invasion in two sites in Alberta, Canada: the University of Alberta Kinsella Research Ranch and the University of Alberta Mattheis Research Ranch (see Stotz et al., 2019 for site descriptions). These sites have brown Chernozemic and Solonetzic soils (Stotz et al., 2016). We revisited nineteen 6 m transects that have been sporadically monitored over 6 years (see Stotz et al., 2019). To measure the rate of invasion, *B. inermis* presence was recorded at every 1 cm along the transect using a line‐intercept method over 6 years (Goldsmith & Sutherland, 1997). A modified belt‐transect method was also used to measure gradual changes in plant community characteristics of invaded areas (Grant et al., 2004). Along the transects, we established ten adjacent 50 × 50 cm sampling plots extending from one transect end (previously invaded, along the invasion front, and into adjacent uninvaded grassland) for a total of 200 sampling plots per site (Figure S1). In this design, the invasion front—but not the plot locations—changes over time, such that different plots along the transect will have been invaded for different durations. We resampled transects and plots in July 2019 to collect current community estimates, abiotic data, and soil samples.

For any plots where *B. inermis* was present before transects were established, we estimated the time since *B. inermis* invasion by modeling the rate of *B. inermis* expansion along each transect using long‐term *B. inermis* abundance data (Stotz et al., 2019) using linear, quadratic, and logarithmic models. We selected the model with the best fit using Akaike's Information Criterion (AIC; Table S1) to interpolate the date of *B. inermis* invasion for each transect. *R* ^2^ values of the best‐fitting models ranged between .776 and .999 (Table S1). In six of the 19 transects, *B. inermis* had completely invaded all points along the transects such that we could not estimate the time since invasion. After these exclusions, our sample consisted of the estimate of time since invasion for a total of 136 plots on 13 transects. Our estimates of time since invasion are coarse but the best time data obtainable at this scale of study, do generate meaningful patterns (see results), and this temporal aspect of understanding invasion is largely missing in the literature.

### 2.3 Changes in *B. inermis* PSFs and vegetative growth

To determine if the strength and direction of *B. inermis* conspecific PSFs changed over time, we collected soil from beneath the clipped vegetation at each sampling plot in 2019. We collected four 20 cm deep soil cores per plot. Soils were sieved over a 4 mm diameter sieve and packed separately to use as a living inoculum in a greenhouse experiment. We sterilized sieves and tools in between plots. Soils were stored at 4°C until used in the greenhouse experiment (within 30 days of collection). Soils among invaded and uninvaded areas are not fundamentally different; thus, with replication and comparisons to non‐invaded plots, we believe we can isolate the effect of soil biota on *B. inermis* growth.

To investigate the contributions of soil biota vs. abiotic factors to *B. inermis* conspecific PSFs, we grew *B. inermis* in three soil treatments. All plants were grown in 340 mL pots, differing in the soil treatment as follows:

1. *Inoculum*: We mixed autoclaved background soil (sterilized coarse sand: fine sand: topsoil mixed in bulk in a 2:1:1 ratio) with field‐collected soil in a 9:1 (306 mL sterilized background soil: 34 mL field soil) ratio to highlight the effect of soil biota (i.e., microbes and soil microfauna) on *B. inermis* performance (Bever, 1994; Brinkman et al., 2010).
2. *Sterile inoculum*: To isolate the effect of abiotic soil properties (including allelochemicals) on *B. inermis* performance, we autoclaved field soil to kill soil biota and then mixed in the 9:1 ratio described above (306 mL background soil: 34 mL sterilized field soil).
3. *Sterile background soil*: Soil in the sterile background soil treatment was autoclaved background soil (the mixture of coarse sand, fine sand, and top soil described above). This treatment is a control to compare the effects of soil biota and field abiotic soil properties on *B. inermis* growth.

We recognize that there are potential side‐effects from autoclaving soil, including increased plant‐available nutrients when compared to non‐autoclaved field inoculum (see Salonius et al., 1967). This could have resulted in increased seedling growth in sterilized vs. non‐sterilized soils. However, autoclaving was the only sterilization method available to us at the time of the experiment and the amount of sterilized soil added to pots was small, so we expect this effect, if present, to be negligible. Soil from each of the 136 field plots was tested separately, allowing us to directly relate greenhouse results to plot‐level field patterns. We grew plants in a blocked design including one inoculum and one sterilized inoculum replicate from each sampling plot in every block, as well as 12 sterile background soil controls per block. This resulted in 852 pots in total (136 sampling plots × 2 soil treatments + 12 sterile background soil controls × 3 replicate blocks). The 12 sterile background soil controls were averaged to find the mean block‐level value. *B. inermis* seeds from a local seed supplier (Gold Medal Seeds, Brooks, AB Canada) were sown directly in pots (i.e., not surface sterilized) and thinned to one individual per pot after 14 days (see Chagnon et al., 2018). For the care of all pots, we followed common horticultural practices which included watering ad libitum. After 15 weeks, we harvested *B. inermis* shoot and root biomass, then washed roots and dried shoot and root biomass at 70°C for 48 h before weighing the samples. Biomass was then used to estimate PSFs for each field plot.

To quantify the strength and direction of abiotic conspecific PSFs for *B. inermis* (PSF_abiotic_), we compared the growth of plants grown in sterile background soil and sterilized inoculum using the formula:

(1) $${\mathrm{PSF}}_{\text{abiotic}}=\ln\left[\frac{B.\;\text{inermis}\;{\text{biomass}}_{\text{sterilized inoculum treatment}}}{B.\;\text{inermis}\;{\text{biomass}}_{\text{sterile background soil}}}\right]$$

To determine how soil biota affects *B. inermis* performance, we compared the growth of plants grown with live inoculum to those grown in sterile background soil (Brinkman et al., 2010; Petermann et al., 2008), using the formula:

(2) $${\mathrm{PSF}}_{\text{biotic}}=\ln\left[\frac{B.\;\text{inermis}\;{\text{biomass}}_{\text{inoculum treatment}}}{B.\;\text{inermis}\;{\text{biomass}}_{\text{sterilized inoculum treatment}}}\right]$$

For both measures, a value of zero represents an absence of a PSF, positive values represent a positive PSF, and negative values represent negative PSFs.

To identify if changes in conspecific PSFs are a function of soil biota or abiotic soil properties, we ran separate LMMs using PSF_abiotic_ and PSF_biotic_ as response variables. To identify if the strength and direction of PSFs change over time, we included time since invasion (Table S1) as the explanatory variable. Field transect nested within site and greenhouse block were specified as random effects. Only field plots where *B. inermis* was present were used in the models to estimate how *B. inermis*‐induced PSFs changed over time. LMMs were fit using the lmer function in the lme4 package (Bates et al., 2020) in program R (v 4.2.1, R Core Development Team, 2022). For all LMMs, data were tested for normality and heteroscedasticity using visual techniques and the Shapiro‐Wilks test.

To assess how conspecific PSFs were associated with *B. inermis* abundance in the field, we estimated *B. inermis* abundance in two ways: density and biomass. First, in 2019, we recorded the number of vegetative *B. inermis* tillers in each plot to estimate *B. inermis* density. We note it is not possible to estimate in the field the number of genetic individuals of this highly clonal species, and thus our measures are estimates of ramet density. Second, we collected *B. inermis* aboveground biomass by clipping plants at the soil surface to estimate its growth. To estimate growth vs. reproductive effort, we separated biomass samples into litter, shoot, and flowering biomass; samples were dried at 70°C for 48 h and subsequently weighed. Reproductive effort was measured as the ratio of the biomass of flowering tillers to total aboveground biomass. We also asked how PSFs affected the probability of *B. inermis* flowering by coding flowers as present or absent. Reproductive effort in *B. inermis* and the number of *B. inermis* tillers were cube‐root transformed before analysis due to their negatively skewed distribution.

To determine the effects of conspecific PSFs on *B. inermis* abundance in the field, we ran two sets of LMMs. *B. inermis* aboveground biomass, litter biomass, number of tillers, reproductive effort, and probability of flowering were response variables in separate models, and PSF_abiotic_ or PSF_biotic_ were explanatory variables (10 models total). Field transect nested within site and greenhouse block were specified as random effects.

### 2.4 Impacts of *B. inermis* invasion on native plant communities over time

To measure shifts in community structure associated with time since invasion by *B. inermis*, we first visually estimated the percent cover of each species present within each sampling plot, then calculated species richness and evenness. Evenness was calculated using the formula:

(3) $$\text{Evenness}=\frac{\text{Species richness}}{\ln\left(-\sum_{i}\ln p_{i}\right)}$$

*p*_*i*_ denotes the proportional abundance of species *i* (note: the denominator of the equation is the Shannon diversity index). We excluded *B. inermis* from these calculations as our focus was on shifts in the abundance of resident species. We also clipped neighboring plants at the soil surface and dried them at 70°C for 48 h to calculate the total biomass of resident species.

To examine how resident community structure changed with the temporal dynamics of conspecific PSFs for *B. inermis*, we used structural equation modeling (SEM) (Grace, 2006; Lamb et al., 2011). This approach allows us to test the a priori hypothesis that the accumulation of negative conspecific PSFs for *B. inermis* may mediate the recovery of resident species diversity through direct and indirect effects. We first developed an initial path model based on prior knowledge of the prevalence of negative conspecific PSF dynamics in the system (Stotz et al., 2018). We used species richness, evenness, and non‐brome biomass as measures of change in resident community structure relative to time since invasion. We standardized data using *z*‐scores (see Table S3 for mean values). We then evaluated model fit to evaluate the support for the theoretical relationships being tested. Models were fit using the lavaan library in R (Rosseel, 2012) and evaluated based on criteria of chi‐squared (χ^2^), comparative fit index (CFI), root mean square error of approximation (RMSEA), standardized root mean square residuals (SRMR), and Akaike's information criterion (AIC) values (Kline, 2010).

## 3 RESULTS

Estimated times since *B. inermis* invasion ranged between 0.02 and 22.42 years and species richness in field plots ranged between 2 and 18 species per plot.

### 3.1 Changes in *B. inermis* PSFs and vegetative growth

**FIGURE 1:**
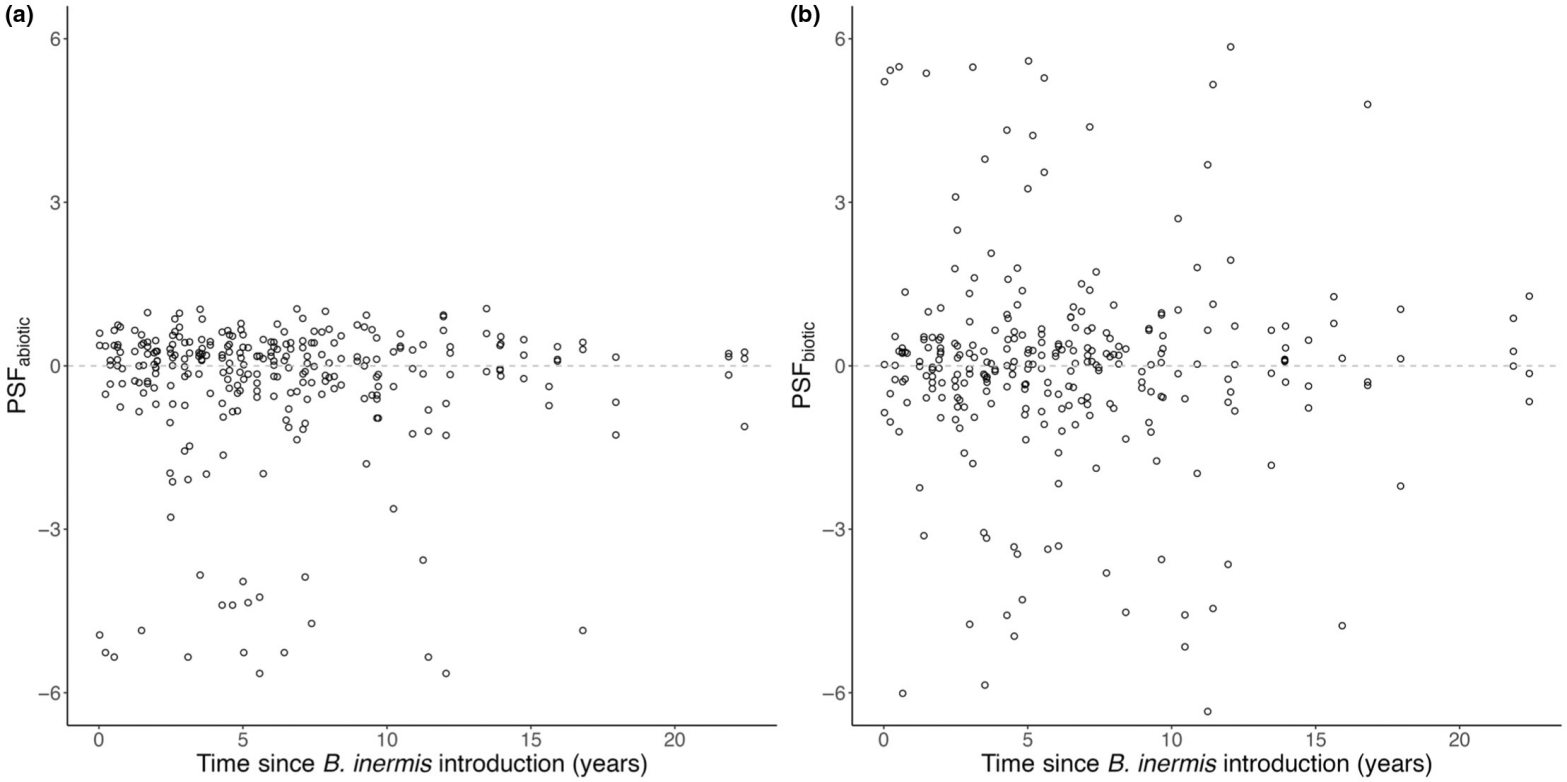
*Bromus inermis* does not incur significant negative PSFs with increasing time since introduction.

PSFs were not significantly positive or negative in the abiotic (PSF_abiotic_: $\chi^{2}$ = 0.19, df = 1, *p* = .66) and biotic PSF treatments (PSF_biotic_: $\chi^{2}$ = 0.22, df = 1, *p* = .64; Figure 1). However, we observed substantial variation in the direction and strength of PSFs, including many negative PSF values (Figure 1).

**FIGURE 2:**
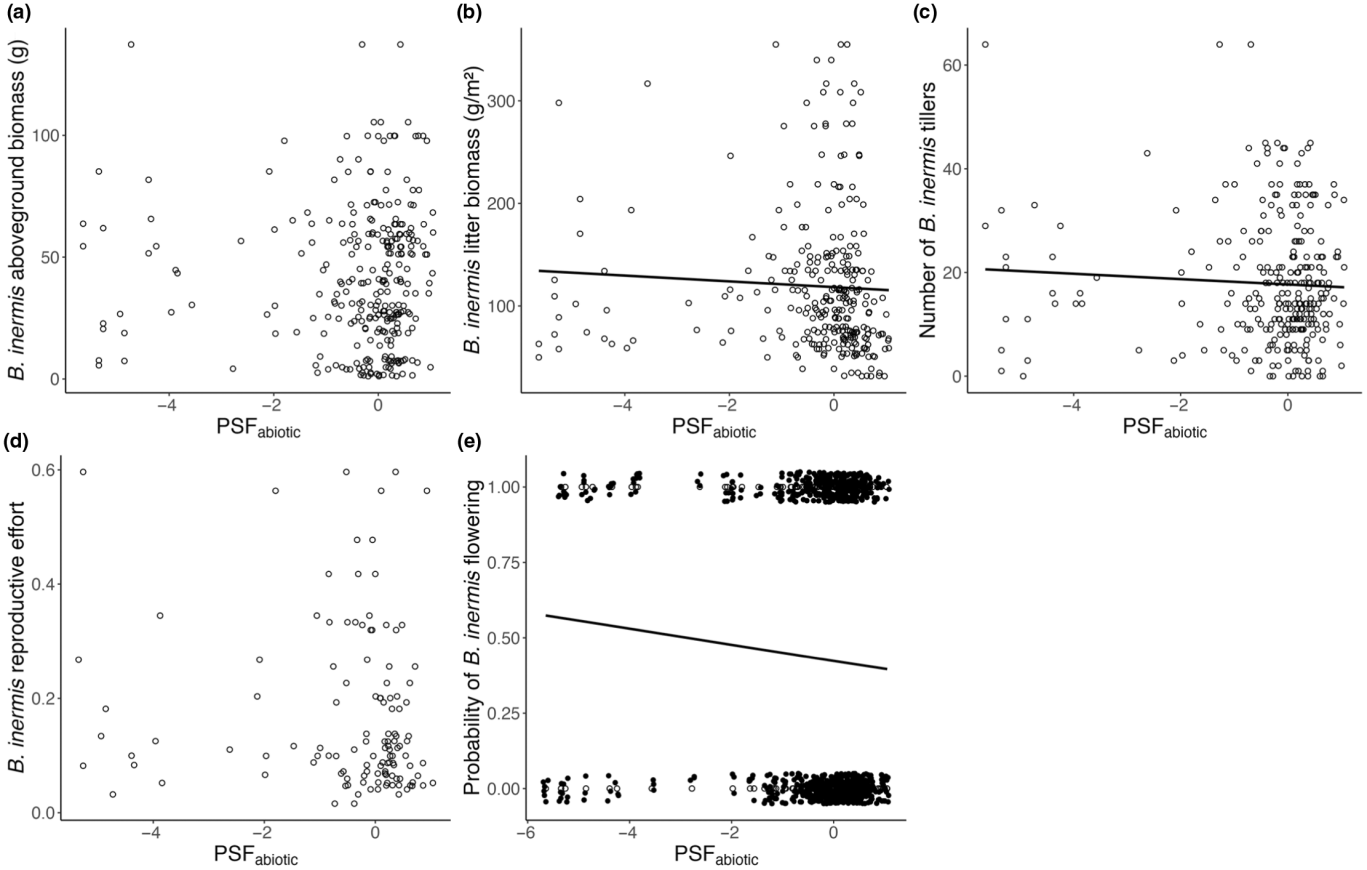
*Bromus inermis* (a) shoot biomass, (b) litter biomass, (c) tiller density, (d) reproductive effort, and (e) probability of flowering as a function of abiotic plant–soil feedback (PSF_abiotic_) strength.

We found little evidence that negative abiotic PSFs reduce *B. inermis* growth in the field, even when such negative PSFs occur. More negative abiotic PSFs resulted in a slight increase in the number of *B. inermis* tillers and an increased probability of *B. inermis* flowering, but did not affect reproductive effort when *B. inermis* was observed to flower ($\chi^{2}$ = 5.72, df = 1, *p* = .02; $\chi^{2}$ = 5.78, df = 1, *p* = .02, and $\chi^{2}$ = 1.15, df = 1, *p* = .28, respectively; Figure 2). Notably, PSFs did not affect shoot biomass in the field ($\chi^{2}$ = 1.22, df = 1, *p* = .27; Figure 2). *B. inermis* litter biomass increased with more negative abiotic PSFs ($\chi^{2}$ = 7.17, df = 1, *p* = .007; Figure 2). PSF_biotic_ were not associated with *B. inermis* density, shoot biomass, litter biomass, or reproductive effort in the field, but the probability of *B. inermis* flowering increased with more positive PSF_biotic_values (Table S3; Figure S2).

### 3.2 Impacts of *B. inermis* invasion on native plant communities over time

**FIGURE 3:**
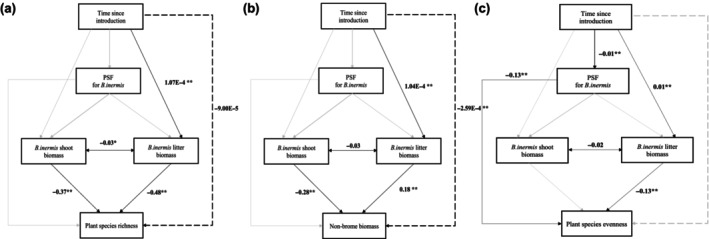
Structural equation models for the long‐term impacts of *Bromus inermis* invasion on (a) plant species richness, (b) non‐brome biomass, and (c) species evenness. Direct relationship from time since establishment and plant community aspect is shown as a solid line, and dashed lines represent indirect mediation effect. Black arrows represent significant relationships (*p‐value <.05, **p‐value <.01), and gray lines represent non‐significant relationships. PSFs in these models are PSF_abiotic_. Standardized path coefficients are shown next to significant pathways.

All SEM models were of good fit based on chi‐square, RMSEA, CFI, SRMR, and AIC values (Table S4). Increased time since *B. inermis* introduction directly decreased resident species richness and non‐brome biomass but did not affect PSFs (Figure 3; Tables S5 and S6). Increased time since *B. inermis* establishment increased litter biomass, indirectly decreasing species richness and evenness (Figure 3a,c; Tables S6 and S5). PSFs did not affect *B. inermis* shoot or litter biomass, resulting in no indirect effects of PSFs on species richness, evenness, and non‐brome biomass (Figure 3). There were also no direct effects of PSFs on resident communities (Figure 3). Instead, *B. inermis* shoot and litter biomass significantly decreased species richness and non‐brome biomass, and *B. inermis* litter biomass decreased species evenness (Figure 3). The effect (path coefficients) of *B. inermis* shoot and litter biomass on plant species richness, non‐brome biomass, and plant species evenness were 1000 times larger than any other effects in the structural equation models (Figure 3; Tables S5–S7).

## 4 DISCUSSION

*Bromus inermis* exhibited neutral to negative conspecific PSFs in greenhouse conditions but these PSFs did not increase in strength over time, suggesting *B. inermis* experiences the full strength of its PSFs upon introduction (Figure 1). *B. inermis* vegetative growth had strong negative effects on resident communities that increased over time: shoot and litter biomass reduced species richness and non‐brome biomass in resident communities but were unaffected by PSFs (Figures 2 and 3). The drivers of *B. inermis'* impact on the resident community were not affected by PSFs (Figures 2 and 3); thus, we find no evidence that conspecific PSFs are effective in limiting *B. inermis* invasion. These findings highlight the importance of studying PSFs in field contexts to identify if and how they function in invasion.

### 4.1 Changes in *B. inermis* PSFs and vegetative growth

*Bromus inermis* did not experience significantly negative conspecific PSFs from its introduction and did not accumulate negative conspecific PSFs over time (Figure 1). This indicates that *B. inermis* dominance is not caused by detrimental soil biota cultured by conspecifics, but its invasion could instead be caused by abiotic factors (see de Kroon et al., 2012; Wei et al., 2020), especially since *B. inermis* is known to substantially change its abiotic environment with increased time since its introduction (Stotz et al., 2019; Vinton & Goergen, 2006). It is important to note that heterospecific PSFs could co‐influence *B. inermis* growth if the resident plant community creates soil conditions beneficial or detrimental to the spread of *B. inermis* (see McCarthy‐Neumann & Kobe, 2010). We encourage future studies to consider how native plants condition the whole soil environment to enhance or limit *B. inermis* invasion. Finally, it is believed that the advantage of invasive species is temporary and should decrease over time (Figure 1; Diez et al., 2010; Dostál et al., 2013; but see also Day et al., 2015). We do not find support for this hypothesis, at least within the time frame of this study.

Abiotic and biotic conspecific PSFs increased *B. inermis* reproduction but not biomass production. Through increasing tillering and flowering, negative PSFs may induce a stress response that shifts energy allocation to reproduction without additional structural investment in reproductive organs (Figure 2; sensu Mooney, 1972). This is adaptive as dispersal through reproduction would remove *B. inermis* genets from the stressful influence of negative PSFs (Reekie & Bazzaz, 2011). Increased reproduction in response to conspecific PSFs could be a potential mechanism underlying *B. inermis*' invasiveness, as increased reproduction increases *B. inermis* spread. Following our observations of increased reproduction, no decline in *B. inermis* populations has been observed in natural communities (Sinkins & Otfinowski, 2012; but see Myhr et al., 1966 for decline when in cultivation). The continued increase in *B. inermis* vegetative growth could be because it increases soil nutrients as it invades, which facilitates its further growth over time (Stotz et al., 2019; Vinton & Goergen, 2006).

### 4.2 Impacts of *B. inermis* invasion on native plant communities over time

After accounting for indirect effects, *B. inermis* conspecific PSFs did not moderate the effects of *B. inermis* on resident community diversity (Figure 3). Instead, native plant richness and evenness declined with increased time since *B. inermis* introduction due to litter accumulation in invaded communities (Figure 3; Table S5; see also Kardol et al., 2007; van der Putten & Peters, 1997). Litter accumulation may have created changes in the abiotic environment associated with increased time since *B. inermis* invasion (Hooper et al., 2000; Zhou et al., 2002), which in turn could exclude some species. Further, *B. inermis* biomass accumulation may cause soil eutrophication through the input of organic substrates (Fierer & Lennon, 2011; Piper, Lamb, & Siciliano, 2015; Piper, Siciliano, Winsley, & Lamb, 2015; Ramirez et al., 2010), potentially decreasing diversity through reductions in niche dimensionality and opportunities for resource partitioning (Harpole & Tilman, 2007).

### 4.3 Management considerations

Decreases in community diversity were driven overwhelmingly by *B. inermis* shoot and litter biomass, not conspecific PSFs. As such, we recommend that conservation actions to mitigate the negative effects of *B. inermis* on native plant communities prioritize biomass removal via herbicide, weeding, or early‐season grazing. For this species, conspecific PSFs do not change over time: we take this as evidence that the nature of PSFs and their cumulative effects are species‐specific. We suggest that if managers intend to use PSF accumulation to limit invasion, PSFs must first be assessed on a per‐species and site basis. Our results further highlight that identifying conspecific PSFs is not enough to warrant intervention: it must be demonstrated that the impact of these PSFs are relevant under field conditions. When PSFs are relevant drivers of invasive species' impact, knowledge of the conditions conducive to negative PSFs could provide useful information to decrease invasive species' growth and facilitate the recovery of the native community. Though PSFs are well‐documented in greenhouse conditions, we echo the calls of others to emphasize that their importance must be validated under field conditions (Forero et al., 2019; Heinze et al., 2016).

The development of conspecific PSFs over time will not be enough to reduce the negative impacts of *B. inermis* invasion, and we are doubtful any microbiome‐based intervention would be useful in this specific context. We conclude that conspecific PSFs do not appear to be an effective management tool to limit *B. inermis* invasion. We encourage others to study the local mechanics of invasion to identify the invasion pathways with the largest effect. This approach will maximize the effectiveness of conservation actions.

## AUTHOR CONTRIBUTIONS

**Emily M. Holden:** Formal analysis (lead); project administration (lead); visualization (lead); writing – review and editing (lead). **Karina Salimbayeva:** Conceptualization (equal); data curation (lead); formal analysis (supporting); investigation (equal); methodology (equal); writing – original draft (lead). **Charlotte Brown:** Formal analysis (supporting); investigation (equal); writing – review and editing (supporting). **Gisela C. Stotz:** Conceptualization (equal); data curation (equal); funding acquisition (equal); investigation (equal); writing – review and editing (supporting). **James F. Cahill:** Conceptualization (equal); funding acquisition (equal); methodology (equal); project administration (supporting); resources (lead); supervision (lead); writing – review and editing (supporting).

## CONFLICT OF INTEREST STATEMENT

None.

## STATEMENT ON INCLUSION

This study was conducted on Treaty 6 territory, which is the traditional territory of Cree, Blackfoot, Métis, Nakota Sioux, Tsuu‐tina, Stoney Nakoda, Iroquois, Dene, Ojibway/Saulteaux/Anishinaabe and homeland of the Métis people. Our study did not incorporate Indigenous peoples and knowledge in its design and execution. We are working to remedy this in our current and future work.

## Supporting information


Data S1

## Data Availability

All data and R scripts are available through the University of Alberta Education and Research Archive via https://doi.org/10.7939/r3‐wmew‐4j53.
