# Supplementary material for: Vegetative growth drives the negative effects of an invasive species on resident community diversity and is not limited by plant–soil feedbacks: A temporal assessment
Source: Ecol Evol. 2024 Jul 22;14(7):e70070. doi: 10.1002/ece3.70070 (PMC11262830; doi:10.1002/ece3.70070)
Supplement: Supplementary file 1 — Data S1 [file ECE3-14-e70070-s001.docx]

**Supporting Information**

**Title:** Vegetative growth drives negative effects of an invasive species on resident community diversity and is not limited by plant-soil feedbacks: a temporal assessment

**Journal name:** Ecology and Evolution

**a**


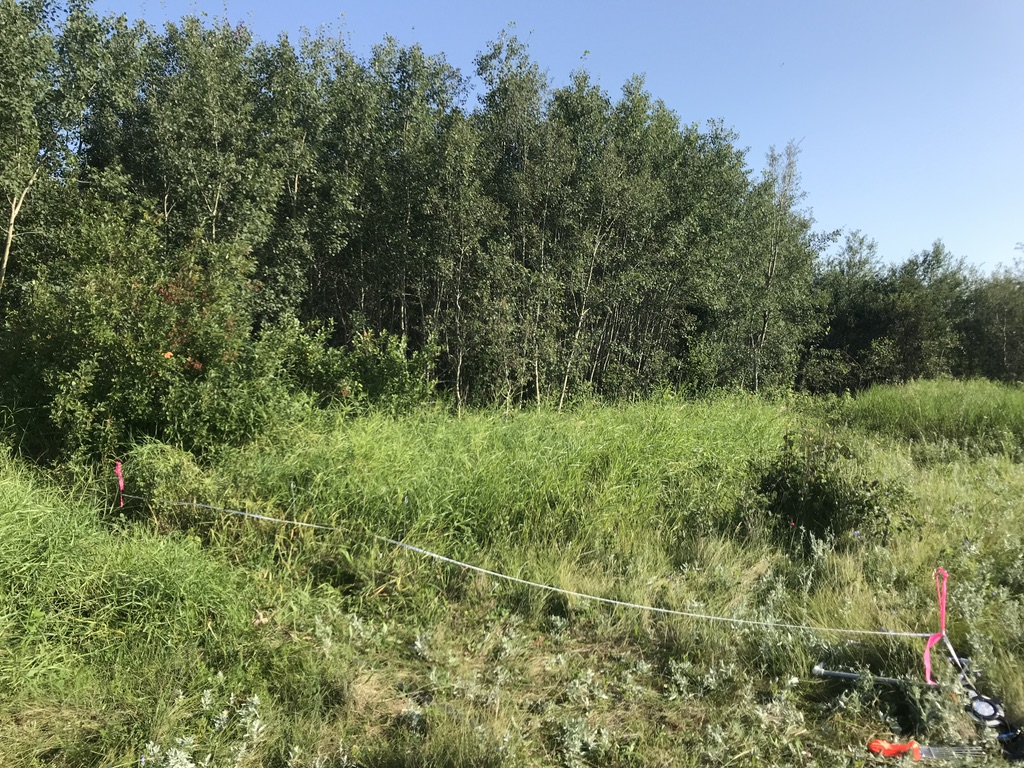


**b**


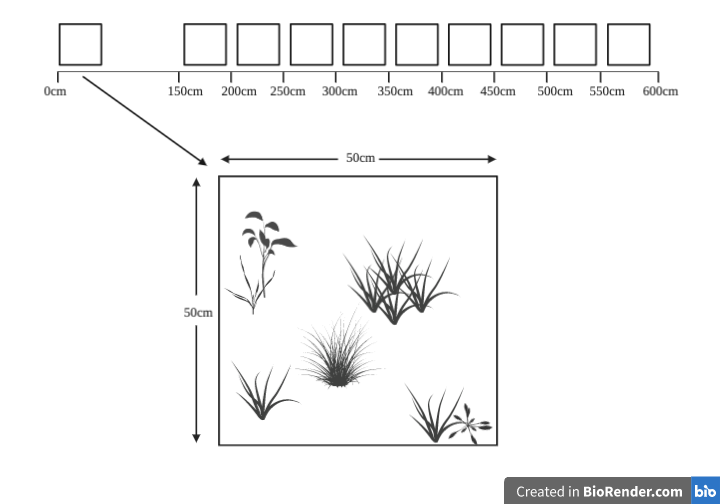


Figure S1: (a) Line-intercept transects were used to encompass the transition between *B. inermis*–invaded areas (blue arrow) to native-dominated areas (red arrow), continuously monitored since 2013. (b) The modified belt-transect method was used to measure resource availability along the transect and community structure (species richness, species evenness and non-brome biomass), and to collect soils to be used as a living inoculum in a greenhouse experiment. Photo credit: Karina Salimbayeva.

Table S1: Model Selection and Akaike's Information Criterion (AIC) to estimate *B. inermis* rate of expansion for each transect. The models examined in this study were: linear (y_i_=𝛽_0_+𝛽_1_X_i_+𝑒_i_), quadratic (y_i_=𝛽_0_+𝛽_1_X_i_+ 𝛽_2_X_i_^2^+𝑒_i_), and logarithmic (y_i_=𝛽_0_+𝛽_1_ln(X_i_)+𝑒_i_), where the dependent variable was y (time since introduction), the independent variable was *B. inermis* abundance along the transect since 2013, 𝛽_0_ and 𝛽_2_ were the coefficients to be determined, and 𝑒_i_ is random error.

|  |  | Linear | | Quadratic | | Logarithmic | |
| --- | --- | --- | --- | --- | --- | --- | --- |
| Site | Transect | R^2^ | AIC | R^2^ | AIC | R^2^ | AIC |
| Kinsella | 1 | 0.908 | 15.929 | 0.911 | 17.726 | 0.914 | 17.593 |
| Kinsella | 2 | 0.999 | -11.630 | 0.998 | -9.630 | 0.988 | 4.111 |
| Kinsella | 3 | 0.979 | 8.505 | 0.979 | 10.467 | 0.972 | 10.953 |
| Kinsella | 4 | 0.984 | 7.059 | 0.984 | 8.893 | 0.981 | 8.855 |
| Kinsella | 5 | 0.994 | 1.609 | 0.996 | 1.649 | 0.986 | 6.469 |
| Kinsella | 6 | 0.776 | 20.377 | 0.839 | 20.731 | 0.792 | 20.451 |
| Kinsella | 7 | 0.978 | 8.735 | 0.978 | 10.727 | 0.971 | 10.304 |
| Mattheis | 1 | 0.812 | 19.507 | 0.987 | 7.848 | 0.776 | 20.378 |
| Mattheis | 2 | 0.932 | 14.406 | 0.934 | 16.202 | 0.926 | 14.812 |
| Mattheis | 3 | 0.974 | 9.607 | 0.987 | 8.152 | 0.958 | 11.929 |
| Mattheis | 4 | 0.876 | 17.406 | 0.927 | 16.766 | 0.892 | 16.709 |
| Mattheis | 5 | 0.961 | 11.573 | 0.965 | 13.089 | 0.971 | 10.271 |
| Mattheis | 6 | 0.860 | 18.013 | 0.907 | 17.971 | 0.843 | 18.601 |

**
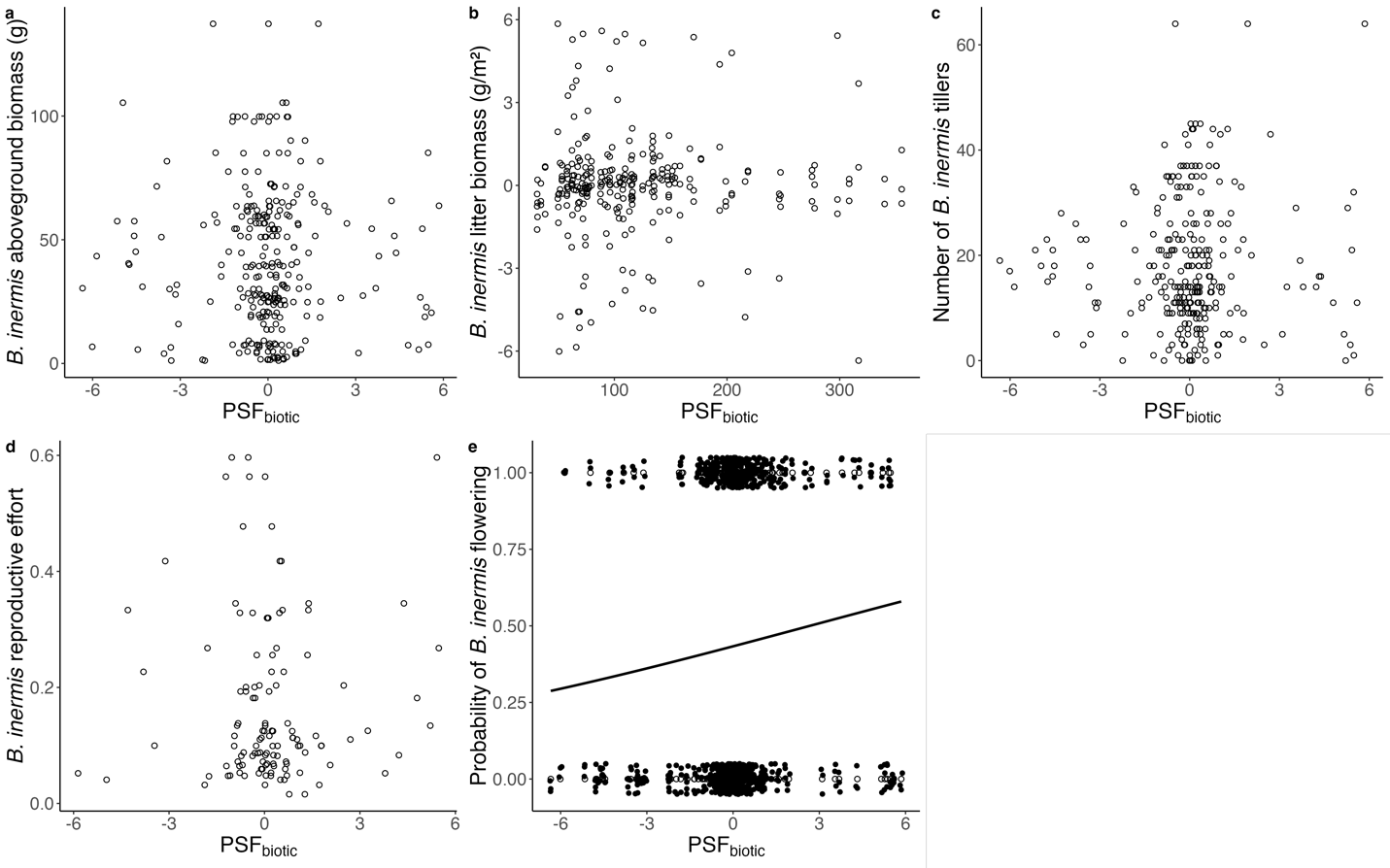
**

Figure S2: *B. inermis* (a) shoot biomass, (b) litter biomass, (c) tiller density, (d) reproductive effort, and (e) probability of flowering as a function of inoculum plant-soil feedback (PSF_biotic_) strength.

Table S2: Linear mixed models results for the effect of PSF_biotic_ on *B.inermis* shoot biomass, litter biomass, tiller density, probability of flowering, and reproductive effort. Bold type and “*” denote a significant relationship.

|  | df | $\chi^{2}$ | p |
| --- | --- | --- | --- |
| Shoot biomass | 1 | 0.21 | 0.65 |
| Litter biomass | 1 | 2.31 | 0.13 |
| Tiller density | 1 | 1.84 | 0.17 |
| Probability of flowering | 1 | 6.49 | **0.01*** |
| Reproductive effort if flowering | 1 | 0.29 | 0.59 |

Table S3: Mean and standard deviation for all variables included in structural equation modeling analysis.

| Variable | Mean ± standard error |
| --- | --- |
| Time since introduction (years) | 6.7 ± 4.78 |
| Plant-soil feedback | -0.48 ± 0.13 |
| *B. inermis* shoot biomass (g) | 518.00 ± 47.60 |
| *B. inermis* litter biomass (g) | 1285.00 ± 120.00 |
| Plant species richness | 6.21 ± 0.77 |
| Plant species evenness | 0.81 ± 0.02 |
| Non-brome shoot biomass (g) | 25.30 ± 3.15 |

Table S4: Chi‐squared values (χ^2^), Comparative Fit Index (CFI), Root Mean Square Error of Approximation (RMSEA), Standardized Root Mean Square Residuals (SRMR), and Akaike’s Information Criterion (AIC) for all SEM models and recommended cut-offs that indicate a good fit.

|  | χ^2^  (p>0.05) | CFI  (≥90) | RMSEA  (<0.08) | SRMR  (<0.08) | AIC |
| --- | --- | --- | --- | --- | --- |
| Plant species richness | 0.862 | 1.000 | <0.001 | 0.005 | -57.281 |
| Plant species evenness | 0.862 | 1.000 | <0.001 | 0.005 | -79.138 |
| Non-brome shoot biomass | 0.862 | 1.000 | <0.001 | 0.005 | -112.680 |

Table S5: Structural equation modeling (SEM) results for total, direct, and indirect effects of *B. inermis* invasion on plant species richness of invaded communities. Standardized estimates are shown, in bold if significant (* p-value < 0.05, ** p-value < 0.01).

|  | **Path Coefficients** | | | | |
| --- | --- | --- | --- | --- | --- |
|  | To plant species richness (SR) | To plant-soil feedbacks for *B. inermis* (PSF) | | To *B. inermis* shoot biomass (SB) | To *B. inermis* litter biomass (LB) |
| Time since introduction (TSI) | **-9.00E-5*** | 2.987E-5 | | 4.590E-5 | 1.069E-4 |
| Plant-soil feedbacks for  *B. inermis* (PSF) | 0.047 |  | | -0.026 | -0.026 |
| *B. inermis* shoot  biomass (SB) | **-0.365**** |  | |  | **-0.026**** |
| *B. inermis* litter  biomass (LB) | **-0.480**** |  | |  |  |
|  | **Indirect effects** | |  | | |
|  | Estimate | Standard error | |  |  |
| Total indirect effect | **-1.226E-4**** | 4.188E-5 | |  |  |
| TSI → PSF → SR | 1.391E-6 | 3.216E-6 | |  |  |
| TSI → SB → SR | 1.676E-5 | 1.522E-5 | |  |  |
| TSI → LB → SR | **-5.136E-5*** | 2.144E-5 | |  |  |
| TSI → PSF→ SB → SR | 2.807E-7 | 6.302E-7 | |  |  |
| TSI → PSF → LB → SR | -3.693E-7 | 8.268E-7 | |  |  |

Table S6: Structural equation modeling (SEM) results for total, direct, and indirect effects of *B. inermis* invasion on plant species evenness of invaded communities. Standardized path coefficients are shown, in bold if significant (* p-value < 0.05, ** p-value < 0.01).

|  | **Path Coefficients** | | | | |
| --- | --- | --- | --- | --- | --- |
|  | To plant species evenness (SE) | To plant-soil feedbacks for *B. inermis* (PSF) | | To *B. inermis* shoot biomass (SB) | To *B. inermis* litter biomass (LB) |
| Time since introduction (TSI) | 0.000 | -0.000 | | 0.000 | **0.009**** |
| Plant-soil feedbacks for  *B. inermis* (PSF) | -0.032 |  | | -0.026 | -0.026 |
| *B. inermis* shoot  biomass (SB) | **-0.230*** |  | |  | **-0.019**** |
| *B. inermis* litter  biomass (LB) | -0.086 |  | |  |  |
|  | **Indirect effects** | |  | | |
|  | Estimate | Standard error | |  |  |
| Total indirect effect | 4.060E-6 | 3.581E-5 | |  |  |
| TSI → PSF → SE | -9.467E-7 | 2.731E-6 | |  |  |
| TSI → SB → SE | 1.056E5 | 1.004E-5 | |  |  |
| TSI → LB → SE | -9.239E-6 | 1.050E-5 | |  |  |
| TSI → PSF→ SB → SE | 1.767E-7 | 4.000E-7 | |  |  |
| TSI → PSF → LB → SE | 6.642E-8 | 1.645E-7 | |  |  |

Table S7: Structural equation modeling (SEM) results for total, direct, and indirect effects of *B. inermis* invasion on non-brome shoot biomass of invaded communities. Standardized path coefficients are shown, in bold if significant (* p-value < 0.05, ** p-value < 0.01).

|  | **Path Coefficients** | | | | |
| --- | --- | --- | --- | --- | --- |
|  | To non-brome shoot biomass (NB) | To plant-soil feedbacks for *B. inermis* (PSF) | | To *B. inermis* shoot biomass (SB) | To *B. inermis* litter biomass (LB) |
| Time since introduction (TSI) | **-0.000**** | 0.000 | | -0.000 | **0.000**** |
| Plant-soil feedbacks for  *B. inermis* (PSF) | -0.009 |  | | -0.026 | -0.026 |
| *B. inermis* shoot  biomass (SB) | **-0.278** |  | |  | **-0.026**** |
| *B. inermis* litter  biomass (LB) | 0.183 |  | |  |  |
|  | **Indirect effects** | |  | | |
|  | Estimate (b) | Standard error | |  |  |
| Total indirect effect | **-0.021** | 0.002 | |  |  |
| TSI → PSF → NB | 0.002 | 0.001 | |  |  |
| TSI → SB → NB | -0.001 | 0.000 | |  |  |
| TSI → LB → NB | 0.000 | 0.000 | |  |  |
| TSI → PSF→ SB → NB | -0.000 | 0.000 | |  |  |
| TSI → PSF → LB → NB | -0.000 | 0.000 | |  |  |
